# Intrapatient Variability (IPV) and the Blood Concentration Normalized by the Dose (C/D Ratio) of Tacrolimus—Their Correlations and Effects on Long-Term Renal Allograft Function

**DOI:** 10.3390/biomedicines10112860

**Published:** 2022-11-08

**Authors:** Ewa Kwiatkowska, Kazimierz Ciechanowski, Leszek Domański, Violetta Dziedziejko, Jarosław Przybyciński, Andrzej Pawlik

**Affiliations:** 1 Clinical Department of Nephrology, Transplantology and Internal Medicine, Pomeranian Medical University, 70-111 Szczecin, Poland; 2 Deparment of Biochemistry and Medical Chemistry, Pomeranian Medical University, 70-111 Szczecin, Poland; 3 Deparment of Physiology, Pomeranian Medical University, 70-111 Szczecin, Poland

**Keywords:** allograft function, tacrolimus, therapy, intrapatient variability

## Abstract

Tacrolimus, in combination with mycophenolate mofetil and glucocorticoids, is the basis of immunosuppressive therapy after renal transplantation. Tacrolimus intrapatient variability (IPV) and the blood concentration normalized by the dose (concentration/dose ratio, C/D ratio) both have an effect on the function of the transplanted kidney. In this study, we examined whether the metabolism rate affected IPV, whether the C/D ratio value was stable in the long-term follow-up, and whether it could be used for IPV measurements. In addition, our study population was examined for the effect of the C/D ratio and IPV on long-term renal function. The C/D ratio and IPV were examined in 170 patients at appointments held at 3, 6, 12 and 24 months after RTx. The average time post renal transplantation was 70 months. Renal function defined as creatinine concentration at the last appointment was examined. Results: the mean C/D ratio in the study group was 1.63. A negative correlation between the C/D ratio and creatinine concentration at the end of the follow-up was observed. Between the C/D ratio < and ≥1.63 groups, significant differences in creatinine concentration at the last appointment were found. No relationship was identified between the mean C/D ratio and IPV. The C/D ratio values increased significantly over a longer post-transplant period (12, 24, 60 and 120 m). We did not find a correlation between the mean IPV and the creatinine concentration from the last appointment. Our study group was divided into terciles according to IPV, while no renal graft function differences were found at the same appointment. Conclusion: the C/D ratio is useful for assessing the effects of the metabolism rate of tacrolimus on the long-term renal graft function. The C/D ratio does not affect the IPV value. IPV calculated from variability of the C/D ratio does not influence transplanted kidney function. The C/D changes over time.

## 1. Introduction

Tacrolimus is the calcineurin inhibitor used to prevent kidney graft loss [1,2]. Unfortunately, tacrolimus can cause nephrotoxicity, the severity of which depends on the serum concentration (C0) of tacrolimus [3,4,5,6]. Low concentrations of the drug favor acute rejection episodes. Due to its narrow therapeutic range, the stability of its concentration must be monitored throughout the treatment process. IPV, or intrapatient variability, is an indicator of the drug’s concentration variability in the patient. Many authors have shown that a high IPV is a marker of poorer renal graft function, favors acute rejection episodes, and increases the risk of the de novo development of DSAs [7,8,9]. IPV is measured according to pre-dose (or trough) tacrolimus concentrations (C0) from specific points during the follow-up period. Some authors consider that this calculation method should be an inherent and automatically processed element of the digital results, as its value is indicative of patient adherence. IPV can be assessed based on the C0 concentrations. However, it is known that in the post-transplant period, the dose of the drug is reduced, and so is its concentration, while sometimes higher doses of the drug are administered to patients with a history of acute graft rejection. These situations will increase IPV. In the present work, following in the footsteps of other authors, IPV was calculated using the concentration/does ratio (C/D ratio), or the concentration normalized by the dose [10]. The C/D ratio alone helps determine whether the patient is a rapid or poor metabolizer. In 2014, Tholking et al. introduced a C/D ratio cut-off point for rapid, intermediate and poor metabolizers. They concluded that the metabolism rate of tacrolimus affected short-term and long-term renal graft function [11]. There are different theories explaining how rapid metabolism is able to adversely affect the function of the transplanted kidney. In this study, we examined whether the metabolism rate affected IPV, whether the C/D ratio value was stable in the long-term follow-up, and whether it could be used for IPV measurements. In addition, our study population was examined for the effect of the C/D ratio and IPV on long-term renal function.

## 2. Patients and Study Design

### Patients

One hundred and seventy renal graft recipients who were in the care of the Clinical Department of Nephrology, Transplantology and Internal Medicine, Pomeranian Medical University, Szczecin, Poland, were reviewed. The average post-transplant period was 70 months ME (a minimum of 5 months and a maximum of 218 months). After their surgeries, all the patients received triple immunosuppressive therapy with glucocorticoids, the calcineurin inhibitor tacrolimus, and mycophenolate mofetil. The concentration and dose of tacrolimus were evaluated at appointments held at 1, 3, 6, 12, 24, 60 and 120 months after RTx. Subsequently, the C/D ratio for each of these appointments and the mean C/D ratio for the appointments held at 3, 6, 12 and 24 months after RTx were calculated to determine how quickly the given patient metabolized the drug. The C/D ratio was calculated by dividing the C0 concentration of the drug in ng/mL by the daily dose in mg. In addition, the lowest creatinine concentration (NADIR Crea) and the highest estimated GFR (ZENITH GFR) value reached within 6 months after RTx, as well as creatinine concentration and eGFR from the last appointment, were evaluated. The C/D values determined at appointments held at 3, 6, 12 and 24 months were used to calculate IPV following the formula:

{[(C/Dmean-C/D on 3 m) + (C/Dmean-C/D on 6 m) + (C/Dmean-C/D on 12 m) + (C/Dmean-C/D on 24 m)]/C/Dmean} × 100%

Recipient characteristics such as age, sex, CIT, the numbers of mismatches for different types of HLAs, rejection episodes, viremia BK virus (BKV) and cytomegaloviruses (CMV) were evaluated. Donor age was evaluated, as well. The clinical characteristics of the patients are presented in Table 1 and Table 2.

## 3. Materials and Methods

Serum creatinine levels were determined using an enzyme immunoassay and whole blood levels of tacrolimus were determined routinely in a hospital laboratory. For the analysis, the C0 concentration of tacrolimus was used. The GFR was estimated using the CKD-EPI Formula with the help of the National Kidney Foundation calculator. The patients involved in the study were treated in line with the Declaration of Helsinki and the Declaration of Istanbul. The local ethics committee of the Pomeranian Medical University, Szczecin, Poland, approved the study protocol—KB-0012/23/18 (05FEB2018).

## 4. Statistical Analysis

Statistica 11 (StatSoft, Tulsa, OK, USA) was used for statistical analysis. The Shapiro–Wilk test was used to study the distribution. The Mann–Whitney U test was used to compare the two groups with non-normal distribution. Correlations were studied by means of the Spearman’s rank correlation test. Non-normally distributed data were shown as the median (minimum–maximum). *p*-values were significant if they were <0.05. The C/D ratios determined at individual appointments were compared using the Kruskal–Wallis test for similarities in distributions. The IPV values in the individual terciles were also compared using the Kruskal–Wallis test for similarities in distributions.

## 5. Results

**Figure 1:**
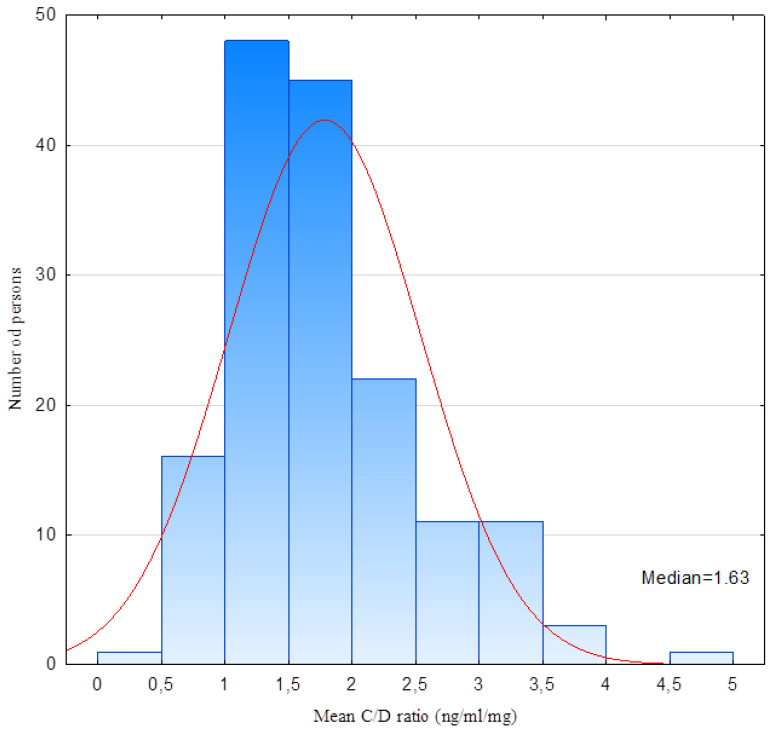
Histogram of the distribution of the C/D ratio (ng/mL/mg). The patients showed non-normal distribution. Median value—1.63.

**Figure 2:**
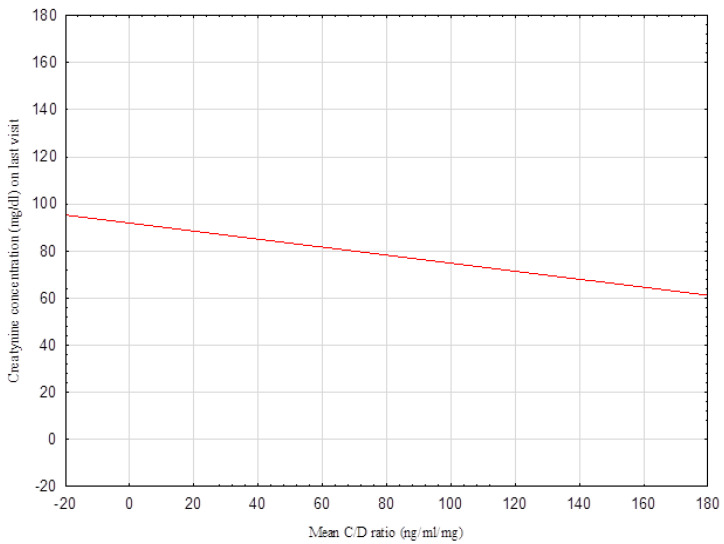
Correlation between the mean C/D ratio and the creatinine concentration from the last appointment. Using Spearman’s rank correlation test, a negative correlation was identified between the mean C/D ratio and the creatinine concentration from the last appointment: *p* < 0.05 R −0.1.

**Figure 3:**
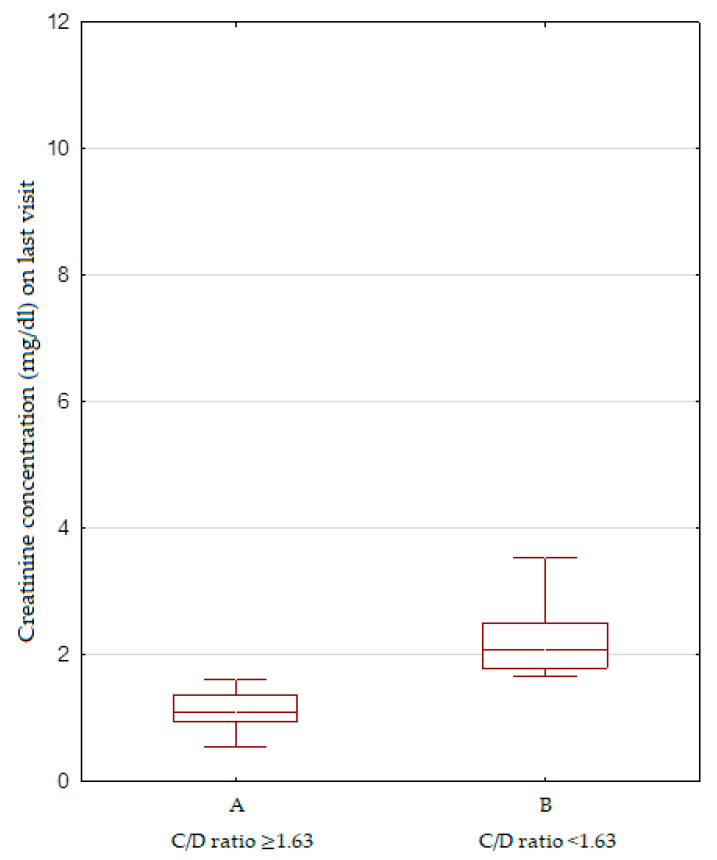
The creatinine concentration from the most recent appointment between the C/D ratio <1.63 (**A**) and ≥1.63 (**B**) groups. Significant differences in the creatinine concentration from the most recent appointment between the C/D ratio <1.63 and ≥1.63 groups (*p* = 0.02, Mann-Whitney U test).

The distributions of the C/D ratio and IPV were calculated using the Shapiro–Wilk test. These distributions were non-normal. The distribution of the C/D ratio is presented in Figure 1. The correlation between the mean C/D ratio and the creatinine concentration from the last appointment is shown in Figure 2. Using Spearman’s rank correlation test, a negative correlation was identified between the mean C/D ratio and creatinine concentration: *p* < 0.05 R −0.1 (Figure 2). The study population was divided into two groups according to the C/D ratio: <1.63 or ≥1.63. There were significant differences between the C/D ratio <1.63 and ≥1.63 groups as revealed by the creatinine levels from the most recent appointments (*p* = 0.02) (Figure 3). No relationship was found between the mean C/D ratio and IPV. IPV did not vary between the C/D ratio <1.63 and ≥1.63 groups, either. We did not find a correlation between the mean IPV and creatinine concentration from the last appointment. Our study group was divided into tertiles according to IPV, while no renal graft function differences were found at the same appointment. We did not find a correlation between the IPV and C/D ratio and data such as: episodes of acute rejection and HLA mismatches, cold ischemic time, number of transplantations, age of recipients and donors; viremia of BKV and CMV. We compared the groups with a C/D ratio above and below 1.63 by using this data, and we did not find any differences between them. We compared the groups divided into tertiles according to IPV and we did not find any differences between them either.

**Figure 4:**
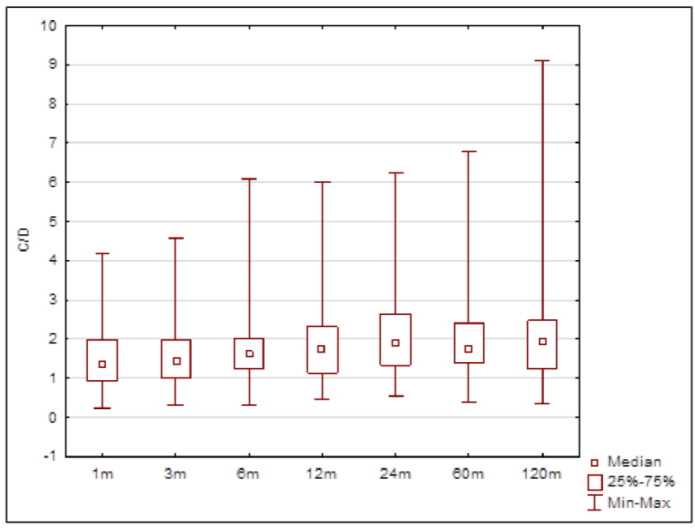
C/D ratio from different points after kidney transplantation. Statistically significant differences are shown in Table 1.

Using the Kruskal–Wallis test, we found that the C/D ratios from individual appointments did not share distribution. Therefore, a post hoc analysis was employed to learn which values differed from others. It was found that the C/D ratio distributions achieved during the first three measurements—at 1, 3 and 6 months—were similar. The distributions from 12, 24, 60 and 120 months differed from the distributions in the first month. The distributions at 24 and 60 months differed from the one at 3 months (the distribution at 120 months lacked significance, although the number of patients observed was smaller). The value obtained at 6 months after RTx had a similar distribution to the distributions determined for points after 6 months (Table 3). The C/D ratios measured in the long-term follow-up period increased (Figure 4).

## 6. Discussion

Tacrolimus is an immunosuppressant commonly used in transplantation that often causes nephrotoxicity, which is dependent on its serum concentration. In daily practice, C0 concentration is used as the only indicator of the metabolism rate of tacrolimus. Maintaining its constant concentration throughout the entire course of therapy is of crucial importance. This is monitored using IPV, or intrapatient variability. As the dose and thus the concentration change with the time after RTx, our study assessed the variability of the C/D ratio, or a ratio of the dose (in mg/day) to C0 concentration, for every patient. IPV from appointments at 3, 6, 12 and 24 months after RTx were calculated. The median IPV was 20%, which meant that at those four appointments, the C/D ratios differed by 20% from the patient mean value. No correlation was observed between IPV and renal graft function at the last appointment, and episodes of rejection or viremia of BKV and CMV. Vanhove et al. found (for a 2-year follow-up after renal transplantation) that higher-IPV patients (the third tertile) had an increased risk of developing advanced histologic lesions in the form of interstitial fibrosis/tubular atrophy (IF/TA) compared with those with low IPV (the low tertile). However, when he analyzed IPV as a continuous variable, he identified no such correlation. IPV had no effect, either as a continuous variable or as individual tertiles, on creatinine concentration and estimated GFR [10]. These authors studied both the variability of tacrolimus C0 concentration and the C/D ratio and found that their effects on chronic lesions in the kidney were somewhat different, hence the conclusion that the variance of these two indicators cannot be used interchangeably [10]. In our paper, we assessed variability over a long period (other publications had follow-up periods of 6–12 months after RTx), hence the need to resort to the C/D ratio instead of the tacrolimus concentration alone, as it drops considerably between 3 and 24 months. One undoubted advantage of our study was that it allowed a long-term evaluation of renal function, where as many as 166 patients were followed up for 60 months and 52 patients were followed for 120 months, and the mean time between renal transplantation and the last appointment was 70 months. High IPV indicates either a period of over- or underexposure to tacrolimus. Overexposure favors the neurotoxic effects of calcineurin inhibitors, while underexposure increases the risk of rejection episodes, which also predispose to fibrosis [3,4,5,6,12]. IPV is affected by many factors. The main cause of high IPV is patient nonadherence. In Borra’s study, patients from the high-IPV quartile were the least adherent [13]. IPV appears to be the best indicator of nonadherence. Whalen et al. evaluated the IPV of the C/D ratio from months 6 to 12 after RTx and found that high-IPV patients had worse renal function expressed as eGFR at 1, 2, 3 and 4 months following the surgery, a higher risk of graft loss and a higher risk of acute rejection within the first year after RTx. In the author’s opinion, deducing IPV from the tacrolimus concentration would have been a mistake. Patients treated for acute graft rejection are prescribed higher calcineurin inhibitor doses, which increases the IPV calculated in this way and offers the erroneous conclusion that higher IPV favors acute rejection episodes. Therefore, he evaluated IPV using—as we did in our study—the C/D ratio [14]. Davis et al. evaluated two indicators: tacrolimus concentration variability and time in therapeutic range (TTR). He found that it was the time the patient stayed outside the therapeutic window that affected acute rejection, graft loss or the development of de novo DSAs, and not IPV alone. He proved that patients with high IPV that remained within the drug’s therapeutic window developed fewer complications compared to those that spent more time outside that window [15]. Perhaps this is the reason for the large disproportions in results obtained in different studies. Most probably, the patients monitored in studies where IPV had an impact on complications of renal graft function spent longer outside the therapeutic window. As mentioned above, high IPV is mostly affected by patient nonadherence. Other factors, such as food, diarrhea or interaction with other drugs, have a contributory role as well. Taking medication during or around a meal reduces their bioavailability. Grapefruits, pomelos, turmeric and ginger have an inhibitory effect on CYP3A activity. By damaging the intestinal mucosa, diarrhea reduces the activity of the *ABCB1* gene coding for P-glycoprotein, an efflux pump that moves the drug out of the cell, which increases tacrolimus bioavailability. Concomitant medications may change tacrolimus absorption, distribution and metabolism. The best-known drugs demonstrating such effects are glucocorticosteroids, calcium-channel blockers, ritonavir, azole antifungals, rifampin and anti-epileptic drugs. The effects of herbal preparations, particularly from St John’s wort, cannot be neglected [16]. Another factor that may affect IPV, although not yet fully explored, is the metabolism rate of drugs. Chung et al. found that the so-called CYP3A5 expressors had lower IPV than CYP3A5 non-expressors. This could indicate that fast metabolizers have lower IPV. The author implicated that the patients with inactive CYP3A5 metabolized the drug mainly by means of CYP3A4, which is very susceptible to induction or inhibition by other factors [17]. Other authors have not confirmed the correlation between CYP3A5 polymorphism and IPV [18,19]. Approx. 95% of Caucasians carry CYT3A5*3, meaning they are non-expressors—having an inactive form of the cytochrome. Despite this, metabolism of tacrolimus demonstrates substantial interpatient variability. It is also influenced by the polymorphism of cofactors of CUP3A4, POR enzymes. Other factors affecting the metabolism of tacrolimus include hematocrit, plasma albumin concentration, age, sex, and body weight. For these reasons, cytochrome expression screening is not useful. Instead, the metabolism rate of the drug should be measured. In 2014, Tholking et al. introduced the C/D ratio, or the ratio of tacrolimus C0 concentration in ng/mL and the daily dose in mg. He also proposed the C/D ratio cutoff points for fast, intermediate and slow metabolizers. Moreover, he found that the rate of metabolism of tacrolimus affected short- and long-term renal graft function [11]. Therefore, in our study we focused on whether the metabolism rate measured as the C/D ratio had an impact on IPV. No correlation was observed between IPV and the C/D ratio. There are no reports available exploring the effect of the metabolism rate of tacrolimus on IPV. It has been shown that the so-called fast metabolizers of tacrolimus have worse long-term renal graft function. The mechanism behind this relationship is not fully understood. It is believed that in order to maintain the recommended C0 level of tacrolimus, fast metabolizers need to first have had a much higher concentration of the drug—the so-called peak level. A high peak level could be associated with calcineurin inhibitor nephrotoxicity. Higher IPV is known to not be responsible for this effect. We found that fast metabolizers of tacrolimus had higher creatinine levels at the end of the follow-up. Our study population was divided into a group with a C/D ratio < the median, and a group with a C/D ratio ≥ the median. The group with a lower C/D ratio (fast metabolizers) had higher levels of creatinine at the end of the follow-up. This confirms other previous reports. Many studies have proven that fast metabolizing patients have worse renal graft function. Our study also aimed at evaluating the C/D ratio over a long-term follow-up period. The ratio was examined at 1, 3, 6, 12, 24, 60 and 120 months. Using the Kruskal–Wallis test, we found that the C/D ratios from individual appointments did not share distribution. Therefore, a post hoc analysis was employed to learn which values differed from others. It was observed that the C/D ratio distributions derived from the first three measurements at 1, 3 and 6 months were similar to each other. The other distributions differed significantly from the results from 1 and 3 months. Subsequently, the C/D ratio grows but its distributions do not differ from each other—as shown in Table 1. An assessment of the C/D ratio from individual points allows for the finding that it increases over time, which means that the metabolism rate of the drug slows down. Kim arrived at similar results in his long-term (5-year) follow-up [20]. Most authors have studied the C/D ratio over a short term after RTx, usually including two or three measurements over 6 months. In our study, and similarly to Kim, we expanded our focus over a longer period. Most likely, the rate of metabolism of tacrolimus decreases as the steroid dose is reduced or discontinued, or as the patient gains weight and ages. One of the significant findings of our study is that the C/D ratio measured during the so-called short follow-up (up to 6 months) cannot be extrapolated to a longer period. A C/D ratio measured after 6 months can be stable in the long-term follow-up. In our study, we decided to use the C/D ratio and examine its variance as we assumed that it would be stable over a longer observation period and not subject to change, contrary to the tacrolimus concentration. Our results show it can be used for assessing IPV only in month 6 after RTx and later. It seems that an IPV measurement based on drug concentration C0 and evaluation of the concentration range would be better.

## 7. Conclusions

IPV measured as a continuous variable does not affect long-term renal graft function, episodes of rejection or viremia of CMV and BKV. As reported by other authors it should be assessed as time in therapeutic range. The time the patient stays outside the therapeutic window affects renal function. Some authors have reported that the genotype associated with tacrolimus metabolism could affect IPV. In our work, we assessed the IPV by assessing the variability of the C/D ratio. The ratio will change if the patient is taking a too low or too high dose, but also when other factors affect the metabolism of tacrolimus. It seems that an IPV measurement based on drug concentration C0 and an estimate of the concentration range would be better. The C/D ratio of tacrolimus invented by Tholking et al. is a useful marker of long-term renal graft function [11]. There is a significant difference between the ratio measured at months 1 to 6 and after 6 months of the post-transplant period, with its value growing. The C/D ratio measured within the first 6 months after RTx does not reflect the metabolism rate in the subsequent stages of the follow-up period. C/D ratio evaluation is indicated in the first 6 months after transplantation and reassessment thereafter. It is difficult to assume an unambiguous value that will allow for division of the patients into slow and fast metabolizers. The median values calculated by us in particular periods may be helpful in this assessment.

## Figures and Tables

**Table 1:** Clinical characteristics of patients.

	N	ME	MIN	MAX
Last Crea (mg/dL)	170	1.24	0.56	9.6
Last eGFR (mL/min/1.73 m^2^)	170	58.5	4.0	107
NADIR Crea (mg/dL)	170	1.18	0.6	5.42
ZENITH eGFR (mL/min/1.73 m^2^)	170	67	11.0	118.0
Time after RTx (month)	177	70.0	5.0	218.0
IPV	148	20.3	0.2	83.0
Mean C/D ratio	158	1.63	0.5	4.5
C/D ratio at 1 mo	138	1.37	0.24	4.2
C/D ratio at 3 mo	139	1.42	0.31	4.59
C/D ratio at 6 mo	147	1.62	0.3	6.1
C/D ratio at 12 mo	141	1.76	0.48	6.0
C/D ratio at 24 mo	141	1.88	0.55	6.3
C/D ratio at 60 mo	126	1.74	0.39	6.8
C/D ratio at 120 mo	52	1.93	0.34	9.1

Last Crea—the mean creatinine level from the last two appointments, Last eGFR—the mean eGFR value (using the CKD-EPI formula) from the last two appointments, NADIR Crea—the lowest creatinine concentration within the first 6 months after RTx, ZENITH eGFR—the highest eGFR within the first 6 months after RTx (CKD-EPI calculation), time after RTx—time after renal transplantation, IPV—intrapatient variability.

**Table 2:** Clinical characteristic of patients.

	N	MEAN	SD
Age of recipients (years)	176	46	12.3
Age of donors	176	45	12
CIT hours	176	21	9
HLA mismatches	176		
A		1	0.7
B		1	0.7
DR		0.75	0.6
Rejection	176	0.16	0.34
Viremia BKV	176	0.08	0.02
Viremia CMV	176	0.09	0.02

CIT cold ischemia time, BKV—BK virus, CMV—cytomegalovirus, viremia was marked as absent—0, present—1.

**Table 3:** The Kruskal–Wallis test and post hoc analysis assessing the differences in C/D ratio distribution between different points after kidney transplantation.

Dependent Variable: C/D Ratio	*p* Value for Multiple (Bilateral) Comparisons; C/D Ratio						
	1 m	3 m	6 m	12 m	24 m	60 m	120 m
1 m		1.000000	0.332275	0.027241	0.000024	0.000151	0.025557
3 m	1.000000		1.000000	0.156188	0.000324	0.001603	0.097722
6 m	0.332275	1.000000		1.000000	0.247089	0.587393	1.000000
12 m	0.027241	0.156188	1.000000		1.000000	1.000000	1.000000
24 m	0.000024	0.000324	0.247089	1.000000		1.000000	1.000000
60 m	0.000151	0.001603	0.587393	1.000000	1.000000		1.000000
120 m	0.025557	0.097722	1.000000	1.000000	1.000000	1.000000	

## Data Availability

The datasets generated and/or analyzed during the current study are available from the corresponding author on reasonable request.
